# Mechanistic insights into phosphorus transformation mediated by *Arthrobacter* and *Sordariomycetes* under long-term high-volume swine manure application in a wheat-rice rotation system

**DOI:** 10.3389/fmicb.2025.1540267

**Published:** 2025-05-13

**Authors:** Chunlong Zhang, Shuang Zhang, Xiaoyan Tang, Bin Zhang, Dejun Liu, Zepeng Yang, Rong Huang, Yingjie Wu, Qi Tao, Youlin Luo, Changquan Wang, Bing Li

**Affiliations:** ^1^School of Pharmacy and Medical Laboratory Science, Ya’an Polytechnic College, Ya’an, China; ^2^College of Resource, Sichuan Agricultural University, Chengdu, China; ^3^Institute of Agricultural Resource and Environment, Sichuan Academy of Agricultural Sciences, Chengdu, China

**Keywords:** functional validation, P related microorganisms, swine manure, soil P fractions, wheat-rice rotation

## Abstract

**Introduction:**

Understanding the impacts of sustained high-input swine manure on soil phosphorus (P), along with identifying and functionally characterizing P-associated microorganisms, can provide a scientific foundation for effective management of soil P in relation to swine manure application. This study provides novel insights into the functional roles of P-associated microorganisms in mediating phosphorus dynamics under long-term excessive swine manure application.

**Methods:**

The study investigated the prolonged impact of high-volume swine manure application on soil P fractions over an 8-year continuous, randomized field trial involving rotating wheat (wet conditions) and rice (flooded conditions) crops. And the soil treated with the prolonged high- volume swine manure application was selected to isolate and identify specific microorganisms, which were subsequently inoculated into soil previously treated with long-term NPK fertilizer (F) and swine manure application (M) for indoor cultivation and functional characterization verification.

**Results:**

The sustained high input of swine manure markedly enhanced soil P activity and microbial P content (*P* < 0.05), specifically extracting P-associated microorganisms, namely *Arthrobacter* sp. M4 bacteria and *Sordariomycetes 2 MS-M4* fungi. Upon separate inoculation of these microorganisms into high-Carbon (C) and high-P soils (M soil, Olsen P > 70 mg kg^–1^, ROC > 150 mg kg^–1^), it was observed that both microorganisms effectively converted available P sources (Ca_2_-P, Ca_8_-P) into organic P reserves through biological immobilization. Conversely, under conditions of low C and low P (F soil, Olsen P < 10 mg kg^–1^, ROC < 75 mg kg^–1^), there was an enhancement in the decomposition and utilization of soil organic C which resulted in increased effective P content via the breakdown of organic phosphates—demonstrating a robust capacity for P transformation. Furthermore, when these phosphate-related microorganisms were introduced to long-term fertilized soils enriched with NPK fertilizer (F), they exhibited a significantly greater enhancement in soil P availability compared to those inoculated into soils subjected to prolonged high inputs of swine manure.

**Discussion:**

The P-related microorganisms *Arthrobacter* sp. M4 and *Sordariomycetes 2 MS-M4* extracted from soils with high P availability were confirmed to have the key functions of enhancing the fixation of inorganic P into organic P (high-C and high-P condition) or promoting the activation of organic P into rapidly available P (low C and low P level). Which may plays an important role in the management of agricultural P nutrients.

## Highlights

• The prolonged application of swine manure enhances the bioavailability of phosphorus in soil.
• Isolation of soil microorganisms, specifically Arthrobacter sp. M4 bacteria and *Sordariomycetes 2 MS-M4* fungi, was performed.
• The isolated microorganisms facilitate the biological fixation of inorganic phosphorus in nutrient-rich environments.
• The isolated microorganisms effectively degrade organophosphorus in nutrient-poor environments.

## 1 Introduction

The rapid advancement of livestock farming has led to a significant increase in the volume of livestock waste (Peng et al., 2015; Guo et al., 2018). However, the effective utilization rate remains approximately 60% (Nobile et al., 2020; Bai et al., 2016), with unused pig manure frequently contributing to environmental issues. The decomposition and incorporation of livestock manure into field represent a crucial method for its resourceful utilization. Swine manure is abundant in P, predominantly in inorganic form (approximately 70%). Many studies have shown that when both short—and long-term swine manure applied to soil as organic compost, it can directly enhance the P content and may induce alterations in soil P fractions, thereby improving P availability (Chen et al., 2022; Zhang C. L. et al., 2024), however, the study of phosphorus components in soil with high amount of swine manure application is still neglected. Concurrently, swine manure contains a lot of organic matter and other mineral nutrients, the application of swine manure induces modifications in the structure of soil microbial communities, which may influence the P cycling processes within the soil.

Soil microorganisms can significantly mitigate the content of soil available P (Olsen P) fixation by converting Olsen P into microbial P (MBP) (Dai et al., 2017). Under certain conditions, they enhance the utilization of unavailable P fractions by solubilizing inorganic P from calcium phosphate and hydroxyapatite (Chen et al., 2019; Qin et al., 2024), as well as various fractions of organic P in the soil through enzymatic activity and other metabolic products (Huang et al., 2019). Long-term application of organic fertilizers can form a more interactive community and enhance the function of microorganisms, thereby increasing the multi-functional potential of soil ecosystems, and may cause microbial functional redundancy (Lin et al., 2019; Sun et al., 2015). The application of swine manure facilitates the transformation of unstable P compounds into bioavailable organic P via soil microorganisms (Chen et al., 2015; Tian et al., 2016) and concurrently reducing soluble inorganic P levels in the soil (González et al., 2019; Lemming et al., 2019). However, an accumulation of recalcitrant organic matter may increase demand for stable organic matter (such as humus), prompting mineralization processes that release active organic P (Tiecher et al., 2017). Thus, introducing swine manure stimulates the proliferation of certain functional soil microorganisms; however, current research predominantly emphasizes short-term studies and low-input scenarios regarding swine manure’s impact on these microorganisms. This may overlooks their feedback mechanisms under excessive input conditions. While a judicious and substantial application of swine manure is advantageous for enhancing resource utilization efficiency.

Consequently, investigating the interaction between functional microorganisms associated with soil P and the availability of soil P from specific P-functional microorganisms holds significant implications for practical applications in agricultural production. Given the dominance of bacterial and fungal taxa in organic P mineralization and inorganic P solubilization (Tian et al., 2016), we focused on isolating and validating these functional groups to elucidate their roles under contrasting nutrient conditions. To clearly suggest the differentiated application of microorganisms in high and low phosphorus soils and enhance the practical guiding significance of the research. In our study, based on an 8-year field experiment involving continuous high-rate application of swine manure, assessed soil P content and selected the prolonged high-rate swine manure treatments to isolate, screen, and functionally characterize bacteria and fungi that constitute a substantial portion of the soil microbial community. The cultured bacterial and fungal solutions were subsequently introduced into the soil and incubated in a laboratory setting for functional verification, concentrating on three key areas: (1) elucidating the characteristics of soil P fractions under sustained high-dose swine manure input; (2) selecting P-functional microorganisms and identifying them via the NCBI platform; (3) clarifying the impacts of these selected bacteria and fungi on both soil P availability and transformations within P fractions. We hypothesized that long-term high-volume swine manure application would: (1) shift soil P fractions toward organic forms via microbial immobilization; (2) select for specific P-transforming microorganisms capable of dual-functionality (P fixation vs. activation) depending on soil nutrient status.

## 2 Materials and methods

### 2.1 Long-term field trial

The general situation of the experimental site is described in our previous research by Zhang C. L. et al. (2024). The field experiment commenced in 2012, incorporating two treatment modalities: a single application of NPK fertilizer (F) (The fertilizer application rate was according to the local farmer practices) and swine manure at a rate of 30,900 kg ha^–1^ (M, representing 150% FN and 600% FP), as detailed in Table 1. The field trial site was laid out as a completely randomized block, with each treatment being applied to three random plots. The experimental area employed a wheat-rice rotation planting pattern, with each plot measuring 20 m^2^. To mitigate water and nutrient runoff, the plots were delineated by plastic film barriers. All fertilizers were applied simultaneously prior to transplanting or sowing during the rice season (*Oryza sativa* L., F-498, June-October) and the wheat season (*Triticum aestivum* L., Wheat 863, November-May).

**TABLE 1:** Experimental fertilization application (kg.ha^–1^).

Treatment	Manure	Urea	Superphosphate	KCl	P application (P_2_O_5_)	Nitrogen application (N)	Potassium application (K_2_O)
F	0	776	1250	250	150	360	150
M	30900	0	0	0	900	540	370

The amount of fertilizer application in rice-wheat rotation.

In 2020, soil samples (generating 24 samples) were collected by earth auger from the surface layer (0-20 cm, this depth represents the primary root zone for wheat and rice cultivation and is most responsive to manure-induced changes in P dynamics) of the paddy field during the rice harvest season and the wheat harvest season following 8 years of treatments. The sampling was conducted using a soil auger at five designated points according to the five-point sampling method, and the samples were subsequently mixed utilizing a four-point technique, retaining 1.00 kg of soil for analysis. The collected soil was dried and sieved through 1.00 and 0.149 mm nylon mesh before being sealed and stored.

The concentration of total P (TP) in soil is quantified using the NaOH fusion-molybdenum-antimony complexometric titration method (Lu, 2000). Soil available P (Olsen P) was determined by extraction with 0.5 mol L^–1^ NaHCO_3_ (pH 8.5) and the molybdenum-antimony resistance coloration method (Olsen et al., 1954). Microbial biomass C (MBC) and microbial biomass P (MBP) was determined using chloroform fumigation extraction (Brookes et al., 1984). Alkaline phosphatase (ALPase) activity was determined using the benzene disodium phosphate colorimetric method (Schneider et al., 2000). The primer utilized for sequencing the *phoD*- gene is ALPS-F730/ALPS-R1101 (CAGTGGGACGACCACGAGGT/GAGGCCGATCGGCATGTCG) (Huang et al., 2019). Dissolved organic C (DOC) using an elemental analyzer, and readily oxidized organic C (ROC) using a potassium permanganate oxidation method (Lu, 2000).

Soil inorganic P fractions were extracted in sequence using the methods of Jiang and Gu (1990), which classify the inorganic P present in soil into six distinct categories: dicalcium phosphate (Ca_2_-P), octacalcium phosphate (Ca_8_-P), aluminum phosphate (Al-P), iron phosphate (Fe-P), occluded phosphate (O-P), and decacalcium phosphate (Ca_10_-P). Soil organic P fractions were extracted in sequence using the method of Bowman and Cole (1978), which classify the organic P present in soil into four distinct categories: Labile organic P (LPo), moderately labile organic P (MLPo), moderately resistant organic P (MRPo) and highly resistant organic P (HRPo). Details of P fractions were described in our previous research by Zhang C. L. et al. (2024).

### 2.2 Isolation and functional validation of key P-utilizing bacteria

#### 2.2.1 Isolation and characterization of specific bacterial strains

Prior to microbial isolation, fresh soil samples were homogenized and sieved (<2 mm), with subsamples stored at 4°C for immediate processing. The culture medium employed for the isolation of soil bacteria (and fungi) via the dilution spreading method is a streptomycin-bengal red medium (Martin et al., 2022; Devkota et al., 2024). The specific procedures are outlined as follows:

In a sterile condition, use a sterile spoon to weigh about 5 g of fresh soil from the M treatment and transfer it into a 150 mL triangular flask. Previously, add 45 mL of deionized water and 10 glass beads to the flask, which were sterilized and prepared for utilization. Then seal it with a sealing film, shake it at 200 rpm for 30 min, and use it as the mother liquid to dilute it 10^–2^, 10^–3^, 10^–4^, and 10^–5^ times for plate coating. The coating volume is 200 μL, and it is evenly coated with a sterile glass rod. After sealing it with a sealing film, keep it right side up for 30 min and then place it in a 25°C incubator upside down for 2-3 days for bacterial culture and 4-5 days for fungal culture. During the culture, pay attention to observation and select 10 or so well-separated colonies on the plate. Perform bacterial line-pure culture on the beef extract peptone agar plate using a spatula, and perform fungal line-pure culture on the potato dextrose agar plate using a spatula. The specific method of transmission is to dig out a single small colony with a spatula and transfer it to the center of a new beef extract peptone agar (or PDA) plate culture medium, and then cover it immediately. Note that to prevent cross-contamination, the alcohol lamp flame should be used to sterilize the spatula before and after picking up each colony. Cultivate at 5°C for 5-7 days and perform the next transmission. In this experiment, a total of 23 bacterial strains and 7 fungal strains were isolated.

#### 2.2.2 Microbiological inoculation and cultivation assessment

**FIGURE 1:**
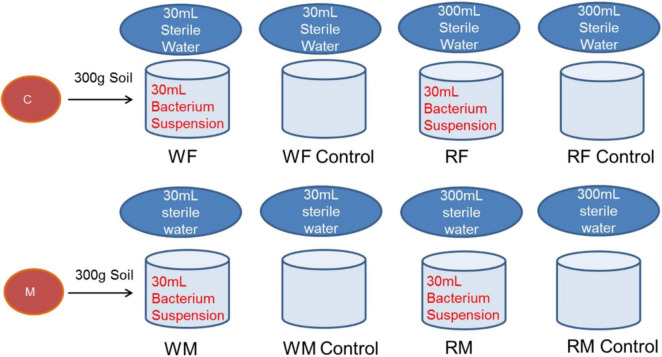
Flow chart of microbial inoculation culture test. The capital letters R and W represent the flooded conditions (rice season) and the wet conditions (wheat season), respectively, the same below. The capital letters F and M on the horizontal axis represent the fertilizer NPK application and the swine manure application, respectively. Bacteria and fungi were cultured separately in beef extract peptone and PDA media, respectively.

After isolating pure strains to a specified number, the resulting strains were cultured in the medium. Four colonies with a diameter of 1 cm (including the medium) were inoculated into 500 mL triangular flasks containing 200 mL of appropriate bacterial beef extract peptone liquid culture medium or fungal PDA liquid culture medium. The cultures were incubated at 25°C with shaking at 200 rpm in constant darkness for a duration of 7 days. Filter the culture solution under sterile conditions, discard the filtrate, and rinse thoroughly with sterile deionized water to ensure that all media components are removed. The colonies were then resuspended in 200 mL of sterile deionized water to obtain a uniform suspension. Inoculation involved adding 30 mL of microbial suspension (10^8^ CFU ml^–1^) to 3,300 g of air-dried soil (F or M treatment) in triplicate. Control groups received sterile deionized water. Detailed operational steps for bacterial and fungal inoculation and cultivation tests are illustrated in Figure 1.

Deionized water was utilized in the experiment to minimize experimental errors. All treatments were incubated in a light-controlled environment for a duration of 6 weeks. The cultivation conditions comprised 14 h of illumination and 10 h of darkness. The temperature during the light phase was maintained at 28°C, while the dark phase was kept at 20°C, with relative humidity consistently held at 70%.

Following the cultivation process, the Olsen P content of each soil sample was assessed, and variations in soil Olsen P content were utilized to further identify and select P-related microbial strains, leading to the isolation of one bacterial strain and one fungal strain. The methodology for determining the Olsen P content is detailed in section 2.1.

#### 2.2.3 Identification of high-quality bacterial strains associated with Olsen P

Classify and identify the strains derived from the soil inoculation experiment. Genomic DNA was extracted using the FastDNA SPIN Kit (MP Biomedicals, United States), following the manufacturer’s protocol. Amplify the extracted DNA using it as a template. Bacterial amplification will be conducted with primers 27F (5′-AGAGTTTGATCCTGGCTCAG-3′) and 1492R (5′-ACGGYTACCTTGTTACGACTT-3′) (Lane, 1991), while fungal amplification will utilize ITS4 (5′-TCCTCCGCTTATTGATATGC-3′) and ITS5 (5′-GGAAGGTAAAAGTCAAGG-3′) (Furan et al., 2022). Compare the resulting sequences against those in the NCBI database to ascertain the classification of the corresponding strains.

#### 2.2.4 Validation of functional characteristics in specific strains

Assess relevant indicators for soil inoculated with selected bacteria and fungi, based on changes in soil Olsen P content. β-glucosidase (β-glu) is chosen as an enzyme activity indicator for the soil C cycle, while alkaline phosphatase (ALPase) serves as an enzyme activity indicator for the soil P cycle (Schneider et al., 2000). Enzyme activities were quantified using a SpectraMax^®^ M5 microplate reader (Molecular Devices, United States) via colorimetric assays (Tiecher et al., 2017). Concurrently, measurements of soil C and P fractions indicators are conducted as outlined in section 2.1.

### 2.3 Statistical analyses

Data normality was verified using Shapiro-Wilk tests and non-normal data were analyzed via Kruskal-Wallis test. Tukey’s *post-hoc* test (*P* < 0.05) was carried out using SPSS 23 (IBM) to test the significance of differences in P related index and fractions across the treatments. Pearson correlation coefficient analysis (*P* < 0.05) was applied to the P related index and fractions. Utilizing DNA sequencing technology and the NCBI data platform for comparative analysis, specific microorganisms were identified, and a phylogenetic tree of these microorganisms was constructed using MEGA X. Phylogenetic trees were constructed using the Neighbor-Joining method in MEGA X with 1000 bootstrap replicates.

## 3 Results

### 3.1 P related index and fractions

**FIGURE 2:**
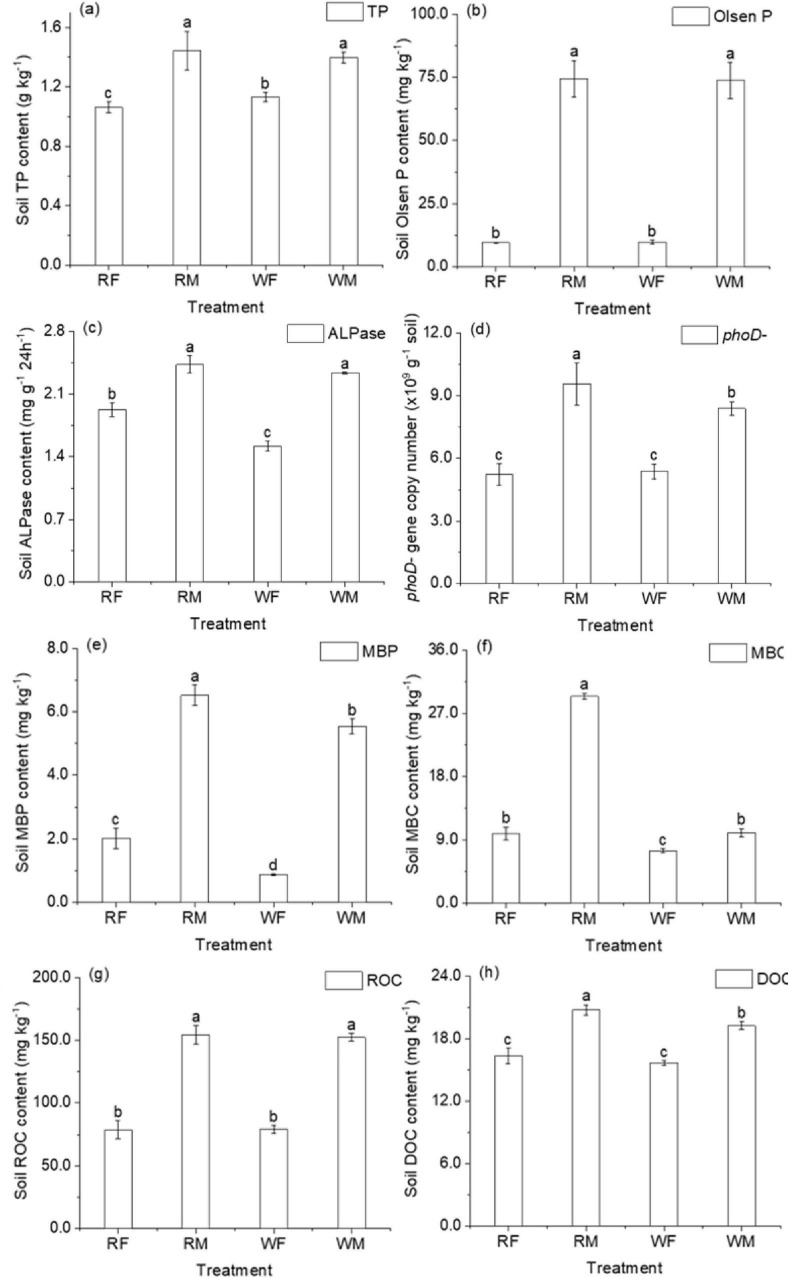
Effects of swine manure returning on chemical properties in the Ultisol. This figure shows the change characteristics of different indexes of soil applied with long-term fertilizer and swine manure, and **(a–h)** were refer to the TP, Olsen P, ALPase, *phoD*-, MBP, MBC, ROC, DOC, respectively. The capital letters R and W on the horizontal axis represent the rice season and the wheat season, respectively, the same below. The capital letters F and M on the horizontal axis represent the fertilizer NPK application and the swine manure application, respectively, the same below. Different letters indicate significant difference among the treatments (*P* < 0.05).

Long-term application of swine manure enhanced the P content and bioavailability in the soil. In comparison to conventional NPK fertilizer application (F), long-term swine manure application (M) resulted in a marked increase in both TP and Olsen P levels, with increases of 35.82 and 683.77%, respectively (*P* < 0.05, Figures 2a,b). These results indicate that sustained high-input swine manure profoundly altered soil P availability. No significant differences were observed in soil phosphorus content between rice and wheat growing seasons under identical treatments. While the application of swine manure notably elevated MBC and MBP levels, the trend for MBP mirrored that of swine manure addition. Furthermore, this practice significantly boosted ALPase activity as well as the abundance of *phoD*- gene copies within the soil, with increases in *phoD*- gene copy numbers surpassing those seen for ALPase activity. The disproportionate increase in *phoD* gene copies (220% higher in M soil; *P* < 0.055) compared to ALPase activity (58% increase; *P* < 0.05) (Figures 2d,e) suggests functional redundancy within the P-cycling microbiome. Thus, it can be concluded that swine manure application substantially augmented populations of P-related microorganisms in the soil; however, their overall metabolic activity remained relatively lower (Figures 2c-f). Additionally, easily available organic C forms such as DOC and readily ROC exhibited significant increases following swine manure applications, with ROC being primarily responsible for this enhancement (Figures 2g,h).

**FIGURE 3:**
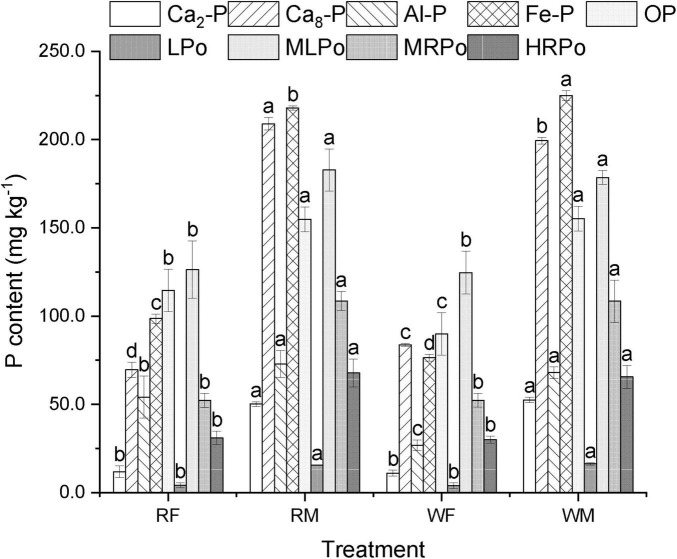
Effect of high-volume swine manure application on soil P fractions. The Ca_10_-P content remain relatively stable, making them the highest among all phosphorus fractions, to highlight the changing characteristics of other phosphorus fractions more effectively, the Ca_10_-P content on the Supplementary Figure 1. Different letters indicate significant difference among the treatments of the same P fractions (*P* < 0.05).

The long-term application of swine manure enhanced the content of various P fractions in the soil and altered their relative proportions (Figure 3). Among the increased P fractions, Ca_8_-P, Fe-P, and MLPo emerged as predominant fractions, notably; Ca_2_-P and LPo exhibited relatively higher increases of 375.15 and 360.71% (WM), respectively. Concurrently, with respect to the distribution of each P fraction, long-term swine manure application markedly elevated the proportions of Ca_2_-P, Ca_8_-P, Fe-P, O-P, LPo, MRPo, and HRPO within the soil matrix. In contrast, stable P fractions (Ca10 -P) remained unchanged, confirming that microbial activity preferentially mobilized bioavailable P pools (Supplementary Figure 1). This indicates a significant enhancement in both active and medium-active P fractions in the soil while improving P bioavailability. The TPi/TPo ratio decreased from 3.86 (RF) to 3.20 (RM), suggesting an increase in organic P proportion within the soil environment due to long-term swine manure application. The observed rise in P bioavailability alongside an increased proportion of organic P may contribute to greater diversity among P-related microbial communities.

### 3.2 Isolation and characterization of microorganisms associated with P

**FIGURE 4:**
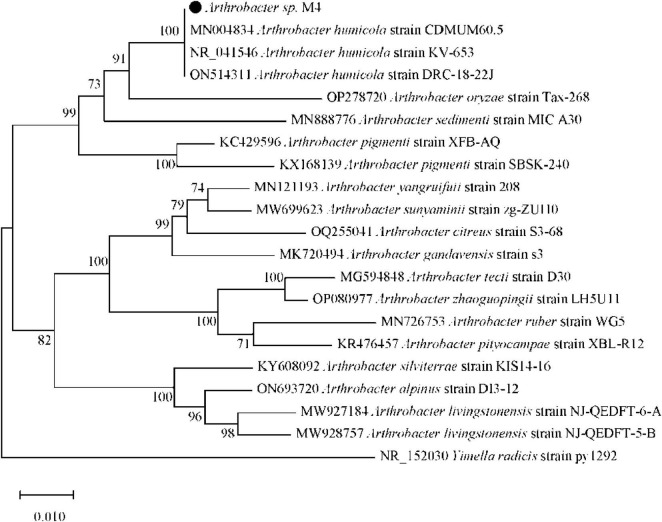
Isolated and screened bacteria 16S rRNA phylogenetic tree. The evolutionary history was inferred using the Neighbor-Joining method, the same as below. The optimal tree with the sum of branch length = 0.47953567 is shown. The percentage of replicate trees in which the associated taxa clustered together in the bootstrap test (1,000 replicates) are shown next to the branches, the same as below. The tree is drawn to scale, with branch lengths in the same units as those of the evolutionary distances used to infer the phylogenetic tree, the same as below. The evolutionary distances were computed using the Maximum Composite Likelihood method and are in the units of the number of base substitutions per site, the same as below. This analysis involved 21 nucleotide sequences. All ambiguous positions were removed for each sequence pair (pairwise deletion option), the same as below. There were a total of 1,548 positions in the final dataset. Evolutionary analyses were conducted in MEGA X.

Select long-term high-rate swine manure input soil for treatment (M), isolate using the dilution plate method, and obtain bacterial and fungal 16S rRNA and ITS sequences through high-throughput sequencing. The bacterial 16S rRNA sequence was assembled to yield a complete length of 1429 bp. Based on this sequence information, the identified species corresponds to a bacterium classified within the *Bacteria*, *Actinomycetota*, *Actinomycetes*, *Micrococcales*, *Micrococcaceae*, *Arthrobacter*, *Arthrobacter* sp. M4. The microbial data is presented in Table 2 and Figure 4.

**TABLE 2:** Information table for identification of P-related bacteria.

Description	Query cover	*E*-value	Per. Ident	Accession
*Arthrobacter* sp. M4 16S ribosomal RNA gene, partial sequence	97%	0.0	99.78%	OR742067

**FIGURE 5:**
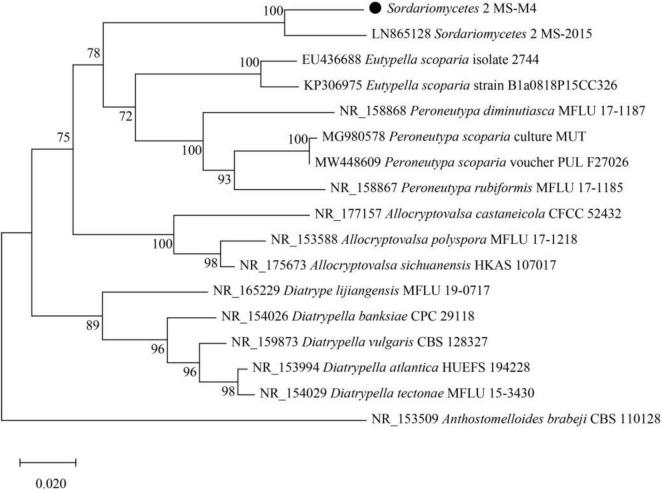
Isolated and screened fungal IFS phylogenetic tree. The optimal tree with the sum of branch length = 0.78737578 is shown. This analysis involved 17 nucleotide sequences. There were a total of 1,451 positions in the final dataset. Evolutionary analyses were conducted in MEGA X.

Upon assembling the fungal IFS sequence, a complete sequence of 588 bp was acquired. Utilizing this sequence information, a comparative analysis with the NCBI database revealed that the extracted sequence was *Eukaryota*, *Fungi*, *Dikarya*, *Ascomycota*, *Saccharomycet*a, *Pezizomycotina*, *Leotiomyceta*, *Sordariomyceta*, *Sordariomycetes*, unclassified *Sordariomycetes, Sordariomycetes 2 MS-M4*. The microbial data is presented in Table 3 and Figure 5.

**TABLE 3:** Information table for identification of P-related fungi.

Description	Query cover	*E*-value	Per. Ident	Accession
*Sordariomycetes 2 MS-M4* genomic DNA sequence contains 18S rRNA gene, ITS1, 5.8S rRNA gene, ITS2, 28S rRNA gene, isolate 2266c	78%	0.0	94.64%	OR742068

### 3.3 Inoculation and cultivation of P-associated microorganisms for functional validation

**FIGURE 6:**
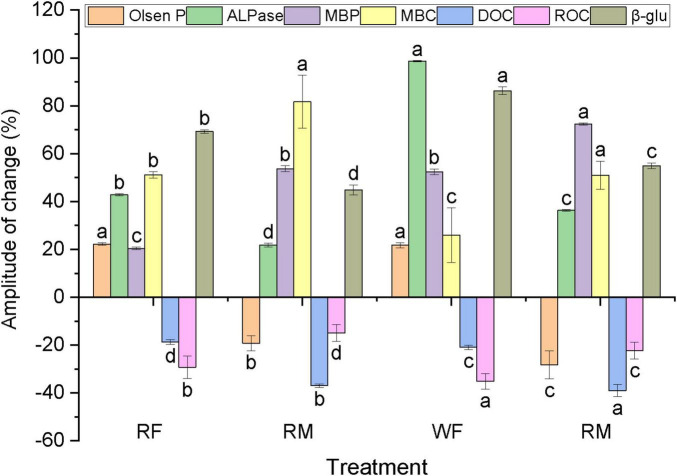
Compared with the control, the change amplitude of the functional characteristics of the soil inoculated by bacteria. In the horizontal axis, R represents flooding treatment in simulated rice season and W represents wetting treatment in simulated wheat season. NPK is the field experiment (2.3.1) NPK fertilizer input (F) treatment, M4 is the field experiment (2.3.1) 900 kg P hm^– 2^ swine manure input, the same below. Different lowercase letters represent the same index with significant difference between different treatments (*P < 0.05*), the same as Figure 7.

**FIGURE 7:**
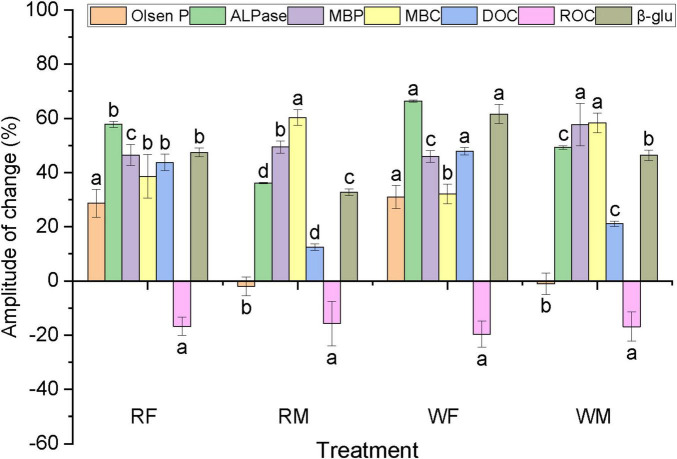
Compared with the control, the change amplitude of the functional characteristics of the soil inoculated by fungi.

The bacterium *Arthrobacter* sp. M4 and the fungus *Sordariomycetes 2 MS-M4*, previously isolated and identified, were individually inoculated into long-term NPK fertilizer-treated soil (F, characterized by low C and low P) and soil enriched with excessive swine manure (M, characterized by high C and high P) for *in vitro* cultivation and functional characteristic assessment, with distilled water serving as the control treatment (Figures 6, 7). Results from the microbial inoculation cultivation experiment indicated that inoculation with *Arthrobacter* sp. M4 significantly enhanced the Olsen P content of the NPK-treated soil, achieving a maximum increase of 8.34 mg kg^–1^(RF), while concurrently reducing the Olsen P content in the M-treated soil by a maximum of 26.24 mg kg^–1^ (WM). Additionally, it markedly increased MBC, MBP, ALPase, and β-glu activity in both NPK- and M-treated soils while decreasing DOC and ROC levels (Figure 6). Overall, the influence of *Arthrobacter* sp. M4 inoculation on NPK-treated soil was more pronounced than on M-treated soil; furthermore, changes in soil indicators under wet conditions were greater than those observed under flooding conditions (Figure 6).

Consistent with the findings from bacterial addition, inoculation with *Sordariomycetes 2 MS-M4* markedly enhanced the Olsen P content in NPK-treated soil, with an increase from 9.69 mg kg–1 to 18.03 mg kg–1 (Figure 7). Control groups (uninoculated soils) showed negligible changes in Olsen P (Δ < 2 mg kg–1) and enzyme activities (Δ < 5% for ALPase and β-glu), confirming that observed effects were driven by microbial inoculation rather than experimental artifacts. For instance, in NPK soil inoculated with S*ordariomycetes 2 MS-M4*, ALPase activity increased by 68% (*P* < 0.01) compared to the control, while β-glu activity rose by 55% (*P* < 0.05), indicating enhanced C-P co-metabolism (Figure 7). In contrast, no significant effect on Olsen P content was observed in M-treated soil. Following the inoculation of *Sordariomycetes 2 MS-M4*, there were substantial increases in soil MBC, MBP, DOC levels, as well ALPase and β-glu activities, conversely, a significant reduction in ROC content was noted. Overall, the impact of *Sordariomycetes 2 MS-M4* inoculation on NPK-treated soil was more pronounced than that on M-treated soil, and variations in soil indicators under wet and flooded conditions remained relatively minor. Furthermore, *Sordariomycetes 2 MS-M4* inoculation demonstrated superior efficacy in enhancing available phosphorus levels within NPK-treated soils compared to *Arthrobacter* sp. M4 inoculation (Figure 7).

The microbial inoculation markedly altered the soil P fractions. With the most significant changes observed in the inorganic P fraction Ca_2_-P and the organic P fractions LPo, MLPo, and MRPo. Notably, the alterations in Ca_2_-P and LPo were pronounced both in magnitude and extent (Figure 7). Both microbial inoculations led to a substantial increase in Ca_2_-P content within NPK-treated soil, specifically, *Sordariomycetes 2 MS-M4* exhibited greater efficacy than *Arthrobacter* sp. M4, achieving a maximum enhancement of 97.71% (WF). Conversely, the M treatment resulted in a significant reduction of Ca_2_-P content by up to 31.52% (bacteria, RM), decreasing it from 50.56 to 34.63 mg kg^–1^. Furthermore, both microbial inoculations significantly elevated LPo levels in the soil, aligning with changes observed in MBP. For LPo specifically, microbial inoculation proved more advantageous for enhancing its concentration in NPK-treated soils; *Sordariomycetes 2 MS-M4* demonstrated a significantly higher increase compared to *Arthrobacter* sp. M4 with an impressive rise of 236.28%, elevating LPo from 4.29 to 14.43 mg kg^–1^. The MRPo fraction exhibited changes solely when microbial inoculation was applied to soils treated with M, both *Arthrobacter* sp. M4 and *Sordariomycetes 2 MS-M4* notably decreased MRPo levels by as much as 18.43% (WF).

The Ca_8_-P fraction exhibited changes solely upon the inoculation of microorganisms into M-treated soil, with these alterations being relatively minor. Inoculation with *Arthrobacter* sp. M4 led to a significant reduction in the Ca_8_-P content within the soil, achieving a maximum decrease of 5.75% in the WM treatment group. Conversely, *Sordariomycetes 2 MS-M4* inoculation resulted in a notable increase in the inorganic P fraction of Ca_8_-P only within the WM treatment; other treatments did not demonstrate any significant impact on soil Ca_8_-P levels. Overall, *Sordariomycetes 2 MS-M4* inoculation had a more pronounced effect on soil P fractions compared to that of *Arthrobacter* sp. M4, particularly evident in NPK treatment soils.

## 4 Discussion

### 4.1 The response of soil phosphorus fractions to prolonged input of swine manure

In this study, the long-term application of swine manure significantly enhanced both the content and proportion of soil active P fractions. This phenomenon can be attributed to the relatively high P content in swine manure (ranging from 0.93 to 5.20%) (Guo et al., 2018) and its predominant presence as orthophosphate post-fermentation (approximately 70%) (Zhang et al., 2021). Consequently, this elucidates the substantial increases observed in TP and Olsen P levels in soil following prolonged swine manure application within this research context. In our study, the average annual total phosphorus content of swine manure in 8 years was 29.23 g kg^–1^, of which inorganic phosphorus accounted for 72.02% and organophosphorus accounted for 17.98%. However, an excessive accumulation of Olsen P may expedite P leaching from the soil, thereby exacerbating the P load on adjacent aquatic environments. Although swine manure increased the proportion of organic P (TPi/TPo decreased from 3.86 to 3.20), this does not imply reduced plant availability. Microbial-mediated conversion of labile inorganic P (Ca2 -P) to microbial biomass P (MBP) and labile organic P (LPo) ensures a slow-release P pool, which reduces leaching risks while maintaining plant uptake through gradual mineralization (Tiecher et al., 2017). This is corroborated by the significant rise in ALPase activity and phoD gene abundance, indicating enhanced organic P turnover capacity. While this study focused on a high swine manure dose (150% N and 600% P of conventional fertilization), the absence of low-to-medium dose treatments limits our understanding of dose-dependent P dynamics. Our prior studies suggest that moderate manure inputs (e.g., 50–100% P requirement) optimize P availability without saturation risks (Zhang C. L. et al., 2024). Therefore, it is imperative to monitor soil P leaching during agricultural practices reliant on long-term swine manure applications and implement strategies aimed at reducing soil P availability—such as converting active inorganic P into organic fractions for stabilization.

Prolonged swine manure application substantially enhanced soil P bioavailability. This improvement may augment the efficiency of soil microorganisms in P utilization, thereby increasing both the diversity and functionality of the soil P microbial community (Lonardo et al., 2019). In this study, the observed increase in organic P proportion within soils subjected to long-term swine manure input can be attributed to microbial activity facilitating the conversion of active inorganic P into biological forms. The notable increases in soil MBP content, ALPase activity, and *phoD-* gene copy number further corroborate this assertion. Notably, when compared to conventional fertilizer applications, the significant rise in *phoD-* gene copy number exceeded that of ALPase activity, indicating functional redundancy among soil microorganisms associated with phosphorus cycling. Despite the benefits of enhanced P availability, prolonged swine manure application may elevate ecological risks. Studies report soil acidification (pH decline by 0.5-1.0 units) and heavy metal accumulation (e.g., Cu, Zn) under high manure inputs (Guo et al., 2018). Additionally, antibiotic resistance genes in manure could propagate in soil microbial communities (Peng et al., 2015). Future research must integrate multi-parameter assessments to balance agronomic benefits with environmental sustainability.

### 4.2 Functional attributes of phosphorus-associated microorganisms in soil

In this study, the selected bacterium *Arthrobacter* sp. M4 is a member of the genus *Arthrobacter* within the phylum *Actinobacteria*, which has been extensively documented in research concerning antibacterial properties (Ait et al., 2023; Baranova et al., 2022; Ebrahimi et al., 2022). In agricultural contexts, it has been reported to synergistically interact with plants to mitigate abiotic stress (Narsing et al., 2022), remediate pesticide residues in agricultural soils (Saez et al., 2022; Giaccio et al., 2023; Emelyanova et al., 2023), fix N, solubilize P and K (Boubekri et al., 2022; Mao et al., 2024), and enhance the solubility of phosphate fertilizers from 2,000 to 2,020, accumulating a total of 949 reports. Consequently, it is frequently utilized as a multifunctional microbial fertilizer following inoculation into fertilizers for agricultural production (Boubekri et al., 2022). Furthermore, studies indicate that this bacterium exhibits heterotrophic (Zhang M. Y. et al., 2020) and autotrophic capabilities (Elkarrach et al., 2021), denitrification processes, degradation of pesticides (specifically Atrazine herbicide) (Zhang et al., 2022), phenol degradation, cold tolerance, and Paccumulation (P removal from wastewater) (Zhang T. et al., 2020). Research also demonstrates that microalgal bacteria exhibit significant responses to exogenous C organic acids addition, leading to marked increases in soil phosphatase activity (Sandra et al., 2020; Nadia et al., 2017). Thus, *Actinobacteria* microorganisms can enhance biological P activity in soil while effectively reducing both P fixation and leaching.

The soil fungal community represents a diverse assemblage within ecosystems, playing a crucial role in various ecological processes and influencing both plant growth and soil health. In this study, the predominant fungal species identified as *Sordariomycetes 2 MS-M4*, extracted from the soil, is classified as a subclass genus of *Ascomycetes*—the most prevalent fungal community found in agricultural soils (Luisa et al., 2024). The medium *Ascomycetes* present in the soil exist in three forms: saprotrophic, parasitic, and symbiotic—interacting with either soil or plant residues—and have been shown to enhance microbial enzyme activity within the soil (Dean et al., 2012; Huang et al., 2021; Xu et al., 2019). The primary metabolic pathway for these fungi involves ATPase catalyzing the hydrolysis of ATP into inorganic phosphate (Luisa et al., 2024; Ondřej et al., 2006). Several studies indicate that *Ascomycete* fungi exhibit a significant positive correlation with available P levels while demonstrating a negative correlation with phosphatase activity (Luisa et al., 2024; Zhang H. et al., 2024). Conversely, Geng et al. (2023) reported that *Ascomycete* fungi were significantly negatively correlated with both Olsen P and TP content, this discrepancy may be attributed to the diversity and functional variability among different species of *Ascomycete* fungi along with their specific roles. Compared to well-known phosphate-solubilizing bacteria like *Pseudomonas* and *Bacillus*, *Arthrobacter* sp. M4 exhibits unique dual functionality—activating organic P in low-P soils while immobilizing inorganic P in high-P soils. This contrasts with *Penicillium* fungi, which primarily enhance inorganic P availability (Correa et al., 2023). However, *Sordariomycetes 2 MS-M4*’s adaptability to flooded conditions surpasses that of *Mortierella* species, highlighting its potential for rice-dominated systems. The dominant fungal species identified herein, along with its secretions, could substantially influence P cycling within the soil ecosystem. To confirm this hypothesis, further investigation is required to elucidate the precise mechanisms involved.

### 4.3 The impact of crucial P-Associated microorganisms on soil P bioavailability

In this study, the bacterium *Arthrobacter* sp. M4, which was isolated and characterized, significantly enhanced soil MBC and MBP content as well as related enzyme activity following its inoculation into the soil (Figure 6). Concurrently, the active organic C components of soil DOC and ROC exhibited a significant decrease, suggesting that the inoculation of *Arthrobacter* sp. M4 improved microbial activity in the soil while markedly enhancing respiration and metabolic processes. These findings align with those reported by Boubekri et al. (2022). Additionally, *Arthrobacter* sp. M4 demonstrated a more pronounced influence on soil indicators under wet conditions than under flooded conditions, indicating heightened activity in aerobic environments. Furthermore, the notable increase in Olsen P content within NPK-treated soils alongside a reduction in Olsen P levels within high nutrient soils (M) suggests that *Arthrobacter* sp. M4 exhibited distinct P functionalities contingent upon varying nutrient levels: it facilitated the conversion of inactive inorganic P to active inorganic P in relatively low-nutrient NPK-treated soils while promoting the fixation of active inorganic P into microbial biomass within high-nutrient soils (M).

The inoculation of the fungus *Sordariomycetes 2 MS-M4* into soil resulted in a significant increase in MBC, MBP content, and ALPase activity, suggesting that the extracted fungi and bacteria may exhibit analogous functional characteristics. This enhancement notably improved soil respiration and microbial activity, contrasting with the outcomes observed when bacterium *Arthrobacter* sp. M4 was introduced into the soil. In this study, while soil ROC content decreased, DOC content increased (Figure 7), potentially due to the preference of *Sordariomycetes 2 MS-M4* for decomposing and utilizing high molecular weight organic matter. A related investigation demonstrated that Mortierella elongata significantly enhanced neutral phosphatase activity in soil while reducing Olsen P levels (Li et al., 2021), which aligns with our findings in M soil, however, *Sordariomycetes 2 MS-M4* exhibited superior efficacy. Compared to *Arthrobacte*r sp. M4, *Sordariomycetes 2 MS-M4* displayed greater adaptability with minimal variation in indicators under both flooding and moist conditions, as well as a more pronounced effect on enhancing P availability in NPK-treated soils. Nevertheless, it is important to note that members of the subclass *Ascomycota* may act as plant pathogens leading to rot issues affecting root and stem health (Huang et al., 2021), necessitating further exploration and validation for agricultural applications. The introduction of P-related microorganisms into soils treated long-term with N, P, and K fertilizers yielded a more substantial enhancement of P availability compared to those inoculated into soils subjected to excessive swine manure over extended periods, this indicates that microorganisms sourced from high-P-availability environments possess stronger capabilities for promoting P release within low-P-soil contexts. While this study did not measure oxalate-extractable P or Mehlich-3 P, the observed reduction in Olsen P under high-P conditions (M soil) aligns with the concept of phosphorus saturation. Future studies should integrate DPS (Degree of P Saturation) analysis to quantify threshold P levels where microbial immobilization becomes dominant. Nevertheless, our functional validation demonstrates that microbial inoculation effectively modulates P availability, suggesting their potential role in mitigating saturation-related environmental risks.

**FIGURE 8:**
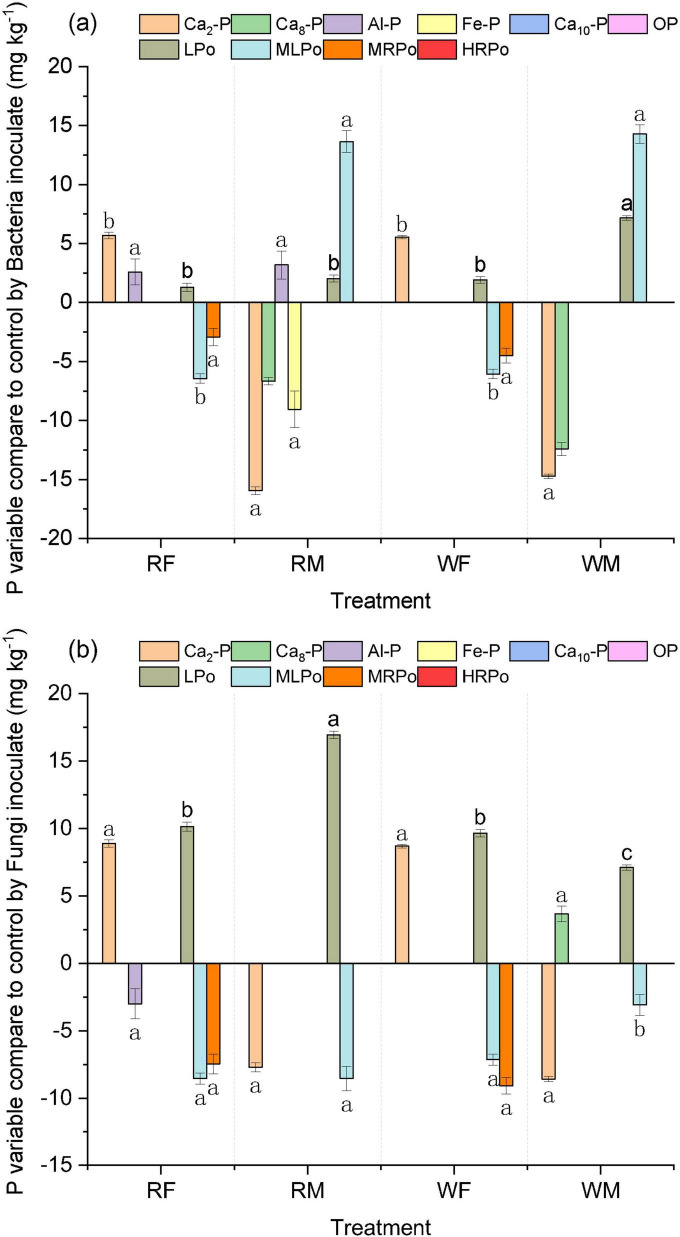
P fractions changes in soil inoculated by bacteria and fungi compared with control. **(a,b)** Show the changes of P components in soil inoculated by *Arthrobacter* sp. M4 and *Sordariomycetes 2 MS-M4*, respectively. Different lowercase letters represent significant difference in the change value of the component under different treatments. We do not show that the treatment has not changed the P fractions.

**FIGURE 9:**
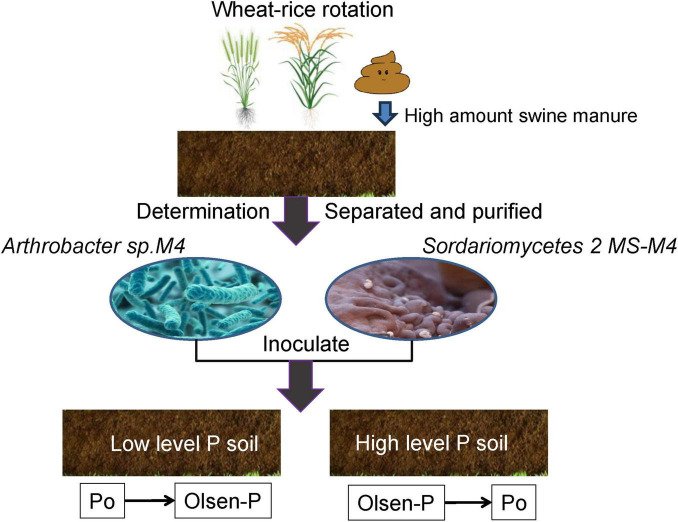
Effect of P solubilizing bacteria isolated from soil by adding excess swine manure on soil P. The P-related microorganisms *Arthrobacter* sp. M4 and *Sordariomycetes 2 MS-M4* extracted from soils with high P availability were confirmed to have the key functions of enhancing the fixation of Olsen P into organic P (high-C and high-P condition) or promoting the activation of organic P into olsen P (low C and low P level).

Microbial inoculation significantly elevated the concentrations of high-activity inorganic and organic P fractions, namely Ca_2_-P and LPo, in the soil, while simultaneously reducing the levels of MLPo and MRPo (Figure 8). This finding indicates that microbial inoculation promotes the interconversion of active P species, which is intricately associated with the energy requirements of soil microorganisms as well as their capacity for decomposing and utilizing soil organic matter (Zhang H. et al., 2024). In contrast to soils characterized by higher C and P content (M), microbial inoculation had a more pronounced effect on C-related enzyme β-glu activity and soil ROC in low C and P environments (F) (Figures 6, 7). This suggests that microbial inoculation enhances both the decomposition and utilization of organic C within low-C, low-P soils (F), thereby facilitating the transformation of organic P into bioavailable inorganic fractions. Consequently, following microbial inoculation coupled with long-term excessive application of swine manure to the soil—resulting in surplus C and P—microorganisms preferentially utilize greater amounts of inorganic P fraction Ca_2_-P while concurrently increasing levels of organic P fraction LPo. Conversely, under sustained application of NPK fertilizers in conditions where C and P are relatively deficient, microorganisms decompose organic P fractions MLPo and MRPo while assimilating available organic matter; this process leads to an increase in both Ca_2_-P and LPo concentrations within the soil matrix. Overall, *Sordariomycetes 2 MS-M4* inoculation exerts a more substantial influence on soil dynamics compared to *Arthrobacter* sp. M4 inoculation; it particularly affects P fractions—especially those that are biologically active—in NPK-treated soils (Figure 9). Translating laboratory findings to field applications requires addressing challenges such as microbial survival under fluctuating temperatures, competition with indigenous microbiota, and scalability of inoculum production. For instance, *Arthrobacte* inoculants showed reduced efficacy in field trials due to predation by protozoa (Boubekri et al., 2022). Pilot studies are essential to optimize formulation and delivery methods for real-world conditions.

## 5 Conclusion

The long-term application of high-volume swine manure significantly enhanced the content and proportion of active P fractions in the soil, while microbial-mediated biological fixation of inorganic P markedly increased the organic P fraction. Specific functional microorganisms involved in P cycling were isolated and characterized from the soil following prolonged high-volume swine manure application. The bacterium *Arthrobacter* sp. M4 and the fungus *Sordariomycetes 2 MS-M4* were identified as key players in this process. Upon reintroducing these P-involved microorganisms into the soil, *Arthrobacter* sp. M4 and *Sordariomycetes 2 MS-M4* effectively utilized substantial amounts of organic C within a high-C, P-rich soil matrix (M), leading to significant reductions in DOC and ROC levels. Consequently, Olsen P concentrations decreased significantly, while MBC, MBP, ALPase, and β-glu notably increased, thus facilitating the conversion of active inorganic P into MBP, thereby enhancing biological fixation processes for this nutrient element. In a low-C environment with limited available P (F treatment), inoculation with *Arthrobacter* sp. M4 and *Sordariomycetes 2 MS-M4* improved utilization rates of soil organic carbon, elevated β-glu enzyme activity along with fluctuations in ROC content, promoted decomposition of organic forms of soil-bound P fractions, converting MLPo and MRPo into more bioavailable forms such as Ca_2_-P and LPo, thereby activating previously unavailable organic P sources to enhance overall nutrient efficacy. The findings suggest that both *Arthrobacter* sp. M4 and *Sordariomycetes 2 MS-M4* possess promising applications for agricultural practices focused on effective P management.

## Data Availability

The datasets presented in this study can be found in online repositories. The names of the repository/repositories and accession number(s) can be found in the article/Supplementary material.
