# Supplementary material for: Mechanistic insights into phosphorus transformation mediated by Arthrobacter and Sordariomycetes under long-term high-volume swine manure application in a wheat-rice rotation system
Source: Front Microbiol. 2025 May 13;16:1540267. doi: 10.3389/fmicb.2025.1540267 (PMC12106499; doi:10.3389/fmicb.2025.1540267)
Supplement: Supplementary file 1 [file Supplementary_file_1.docx]

**FIGURE A1**

Effect of high-volume swine manure application on soil Ca_10_-P. Different letters indicate significant difference among the treatments of the same P fractions (*P*<0.05).
